# Supplementary material for: Close contact infection dynamics over time: insights from a second large-scale social contact survey in Flanders, Belgium, in 2010-2011
Source: BMC Infect Dis. 2021 Mar 18;21:274. doi: 10.1186/s12879-021-05949-4 (PMC7971398; doi:10.1186/s12879-021-05949-4)
Supplement: Supplementary file 1 — Additional file 1 Descriptive data analysis of the 2010-2011 dataset [file 12879_2021_5949_MOESM1_ESM.pdf]

Additional file 1

1 Number of participants by age groups of 5 years and time indicators in the 2006 and the 2010/2011 social contact survey

Table S1: Number of participants by age groups and time indicators. C1 is denoted for regular-weekdays, C2 for holiday-weekdays, C3 for regular-weekend and C4 for holiday-weekend.

	The 2006 Survey										The 2010/2011 Survey				
	The 1st day					The 2nd day									
**Age groups**	**C1**	**C2**	**C3**	**C4**	**Total**	**C1**	**C2**	**C3**	**C4**	**Total**	**C1**	**C2**	**C3**	**C4**	**Total**
$[0, 5)$	9	4	4	35	**52**	5	34	4	9	**52**	81	20	28	17	**146**
$[5, 10)$	16	7	1	29	**53**	1	29	4	19	**53**	74	18	19	11	**122**
$[10, 15)$	14	6	4	21	**45**	4	21	3	17	**45**	38	18	8	6	**70**
$[15, 20)$	21	11	5	16	**53**	5	16	5	27	**53**	36	6	13	8	**63**
$[20, 25)$	16	6	7	4	**33**	7	4	8	14	**33**	48	12	10	3	**73**
$[25, 30)$	12	7	3	9	**31**	3	9	7	12	**31**	74	22	19	9	**124**
$[30, 35)$	12	2	5	6	**25**	5	6	4	10	**25**	72	13	27	12	**124**
$[35, 40)$	13	6	2	6	**27**	3	5	7	12	**27**	82	22	21	10	**135**
$[40, 45)$	14	2	4	3	**23**	4	3	4	12	**23**	88	18	19	16	**141**
$[45, 50)$	24	6	4	3	**37**	4	3	10	20	**37**	95	25	19	11	**150**
$[50, 55)$	22	2	11	3	**38**	11	3	7	17	**38**	101	28	14	17	**160**
$[55, 60)$	23	2	7	1	**33**	7	1	12	13	**33**	38	13	17	6	**74**
$[60, 65)$	18	1	4	5	**28**	4	5	6	13	**28**	47	13	15	8	**83**
$[65, 70)$	8	0	3	4	**15**	4	3	2	6	**15**	42	18	7	6	**73**
$[70, 75)$	6	3	0	0	**9**	0	0	3	6	**9**	40	18	10	3	**71**
$[75, 80)$	4	0	1	1	**6**	1	1	3	1	**6**	32	12	4	5	**53**
$[80, 85)$	1	2	0	0	**3**	0	0	1	2	**3**	18	4	4	2	**28**
$[85, 90)$	0	0	0	0	**0**	0	0	0	0	**0**	14	6	8	2	**30**
$[90, 95)$	0	0	0	0	**0**	0	0	0	0	**0**	12	4	2	1	**19**
$[95, 100)$	0	0	0	0	**0**	0	0	0	0	**0**	13	2	3	0	**18**
**Total**	**233**	**67**	**65**	**146**	**511**	**68**	**143**	**90**	**210**	**511**	**1045**	**292**	**267**	**153**	**1757**

2 Histogram of the number of contacts

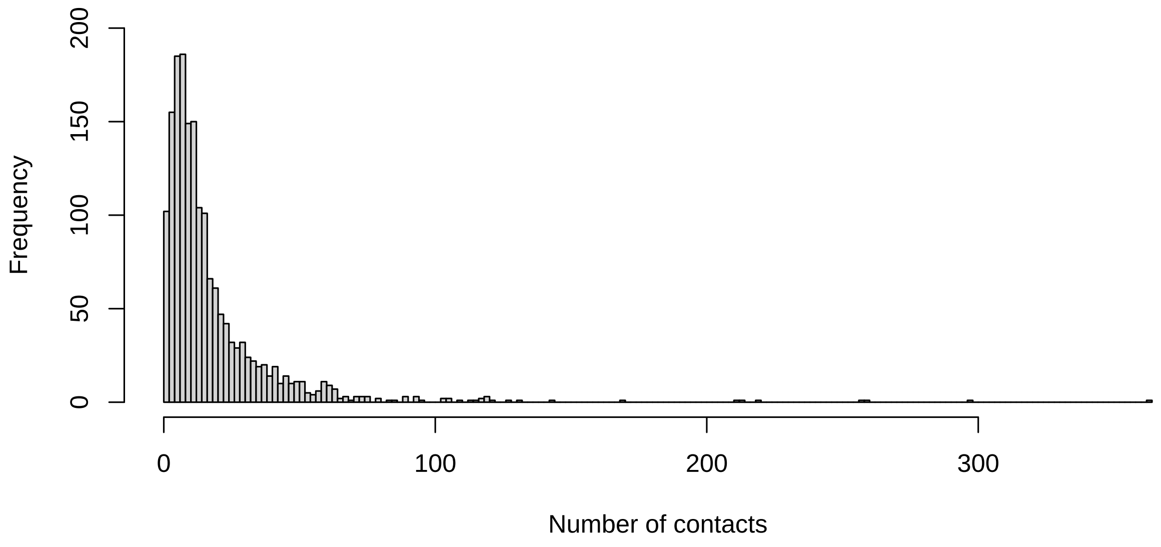

Figure S1: Histogram of the number of contacts on original scale

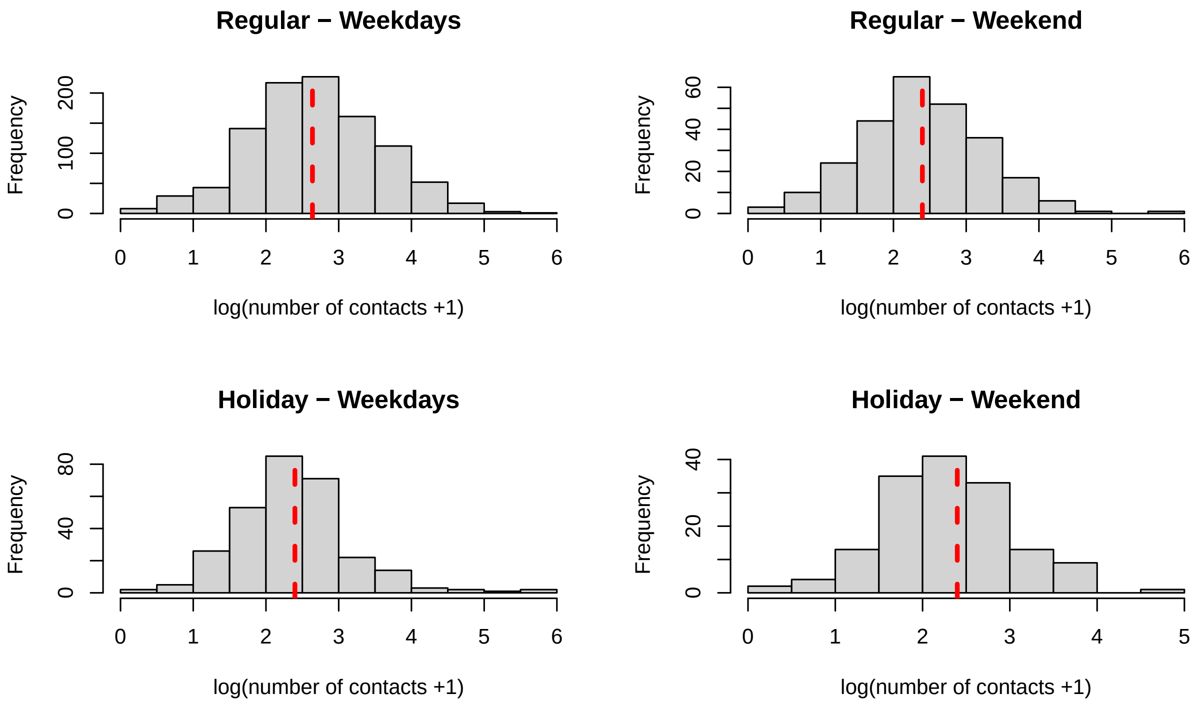

Figure S2: Histograms of log(number of contacts + 1) on weekdays (left) and during the weekend (right), distinguishing regular (top row) from holiday (bottom row) period. The vertical lines present the median values

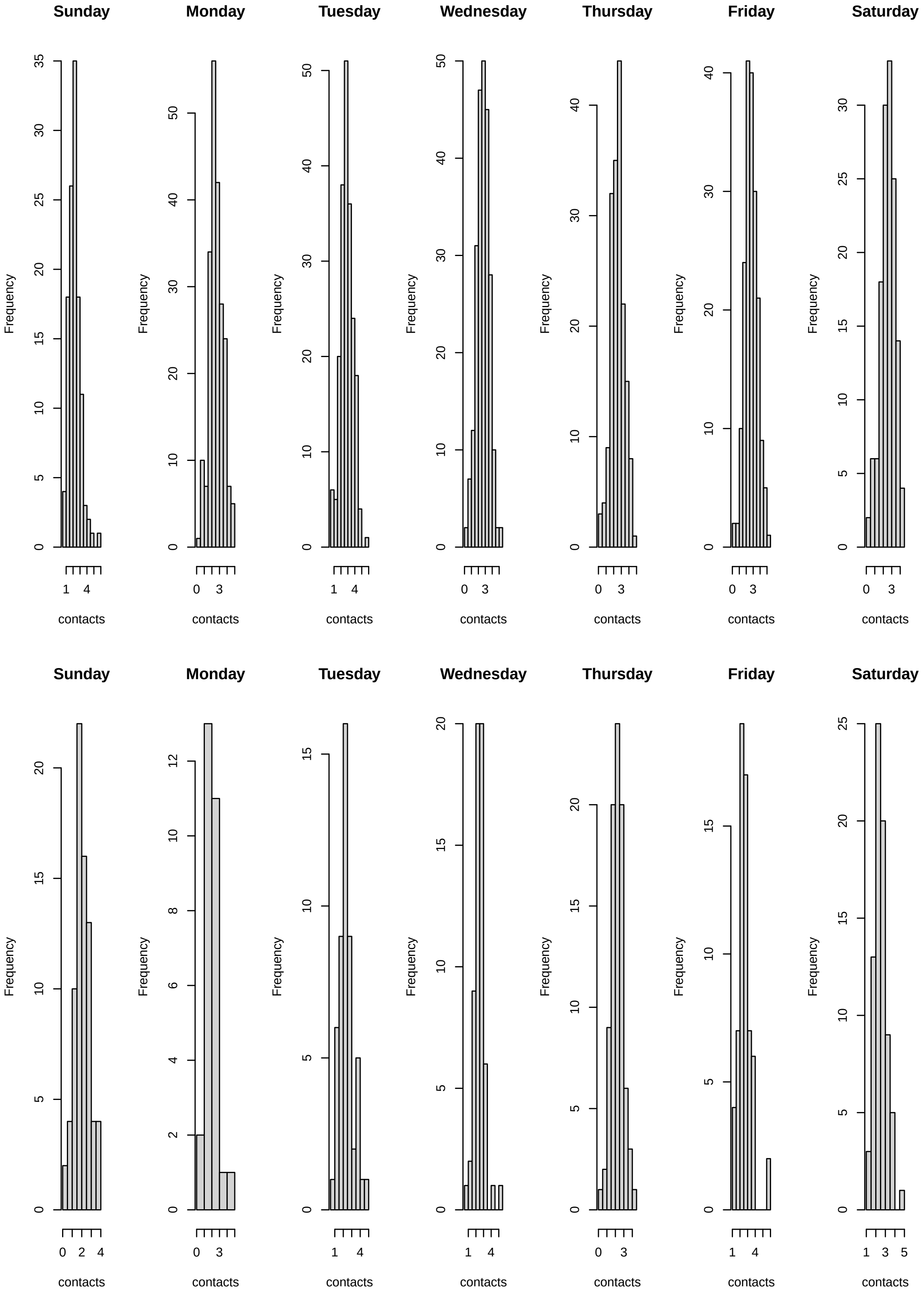

Figure S3: Histograms of log(number of contacts+1) per day in the week, distinguishing non-holiday (top row) from holiday (bottom row) periods.

3 Social-demographic characteristics of participants

Table S2: Frequency table of social-demographic characteristics of participants

Variables	Categories	Label	Number (%) or mean(SD)
**Age**			38.34 (22.46)
**Sex**	Male	M	790 (46.28%)
	Female	F	917 (53.72%)
**Occupation**			
	Working	1	828 (48.51%)
	Retired	2	230 (13.47%)
	at home	3	143 (8.38%)
	unemployed/job seeking	4	45 (2.64%)
	in education	5	360 (21.09%)
	other	6	93 (5.45%)
	Missing	7	8 (0.47%)
**Education**			
	No formal schooling	0	9 (0.53%)
	Primary school	1	118 (6.91%)
	Lower Secondary school	2	177 (10.37%)
	Upper secondary school	3	429 (25.13%)
	secondary school (unspecified)	4	176 (10.31%)
	Lower University degree	5	545 (31.93%)
	Higher university degree	6	242 (14.18%)
	University degree (unspecified)	7	0
	Vocational education (FI)	8	0
	Mising	9	11 (0.64%)
**Household size**			
	1	1	189 (11.07%)
	2	2	497 (29.12%)
	3	3	344 (20.15%)
	4	4	441(25.83%)
	5+	5	228 (13.36%)
	Missing	6	8 (0.47%)
**Child care/school**			
	Stay at home	1	23 (7.42%)
	Day care outside home	2	57 (18.39%)
	Pre-school (2.5-6)	3	92 (29.68%)
	School(6-12)	4	138 (44.52%)
**Day of the week**			
	Sunday	0	194 (11.36%)
	Monday	1	242 (14.18%)
	Tuesday	2	253 (14.82%)
	Wednesday	3	296 (17.34%)
	Thursday	4	259 (15.17%)
	Friday	5	247 (14.47%)

Table S2

Variables	Categories	Label	Number (%) or mean(SD)
Continued previous page			
	Saturday	6	214 (12.54%)
	Missing	7	2 (0.12%)
**Holiday**			
	Yes	Y	437 (25.60%)
	No	N	1268 (74.28%)
	Mising	M	2 (0.12%)

Table S3: Details on occupations of participants

Variables	Catergories	Labels	Frequency (%)
**Occupation**			
**Self-employee**	Craftsman, trader without employee	1	47 (2.75%)
	Craftsman, trader with 5 employees or less	2	0
	Craftsman, trader with 6 employees or more	3	15 (0.88%)
	Free occupation	4	37 (2.17%)
**Office Clerk**	Member of the executive Board, upper level	5	41 (2.40%)
	Middle level	6	200 (11.72%)
	Others	7	471 (27.59%)
**Manual worker**	Vocational-training worker	8	119 (6.97%)
	worker without vocational training	9	80 (4.69%)
**Others**	Housewife	10	121 (7.09%)
	Disabled	11	36 (2.11%)
	Retired	12	26 (1.52%)
	Student	13	130 (7.62%)
	Unemployed	14	45 (2.64%)
	Rentier	15	2 (0.12%)
**Missing**	Missing	16	337 (19.74%)

4 Health indicators and the number of contacts

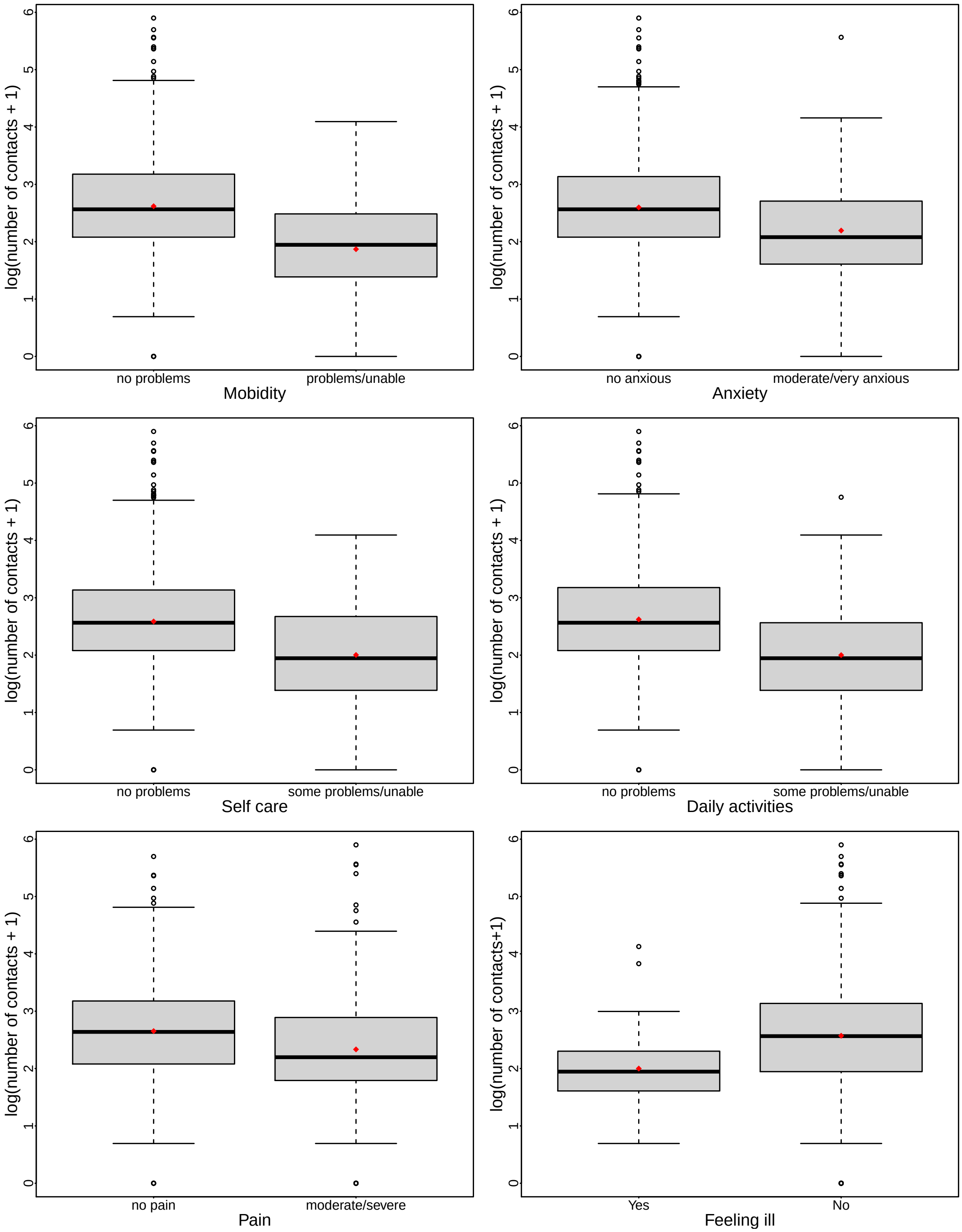

Figure S4: Health indicators and the number of contacts. The red dots represent mean values.

5 Socio-demographic indicators and the number of contacts

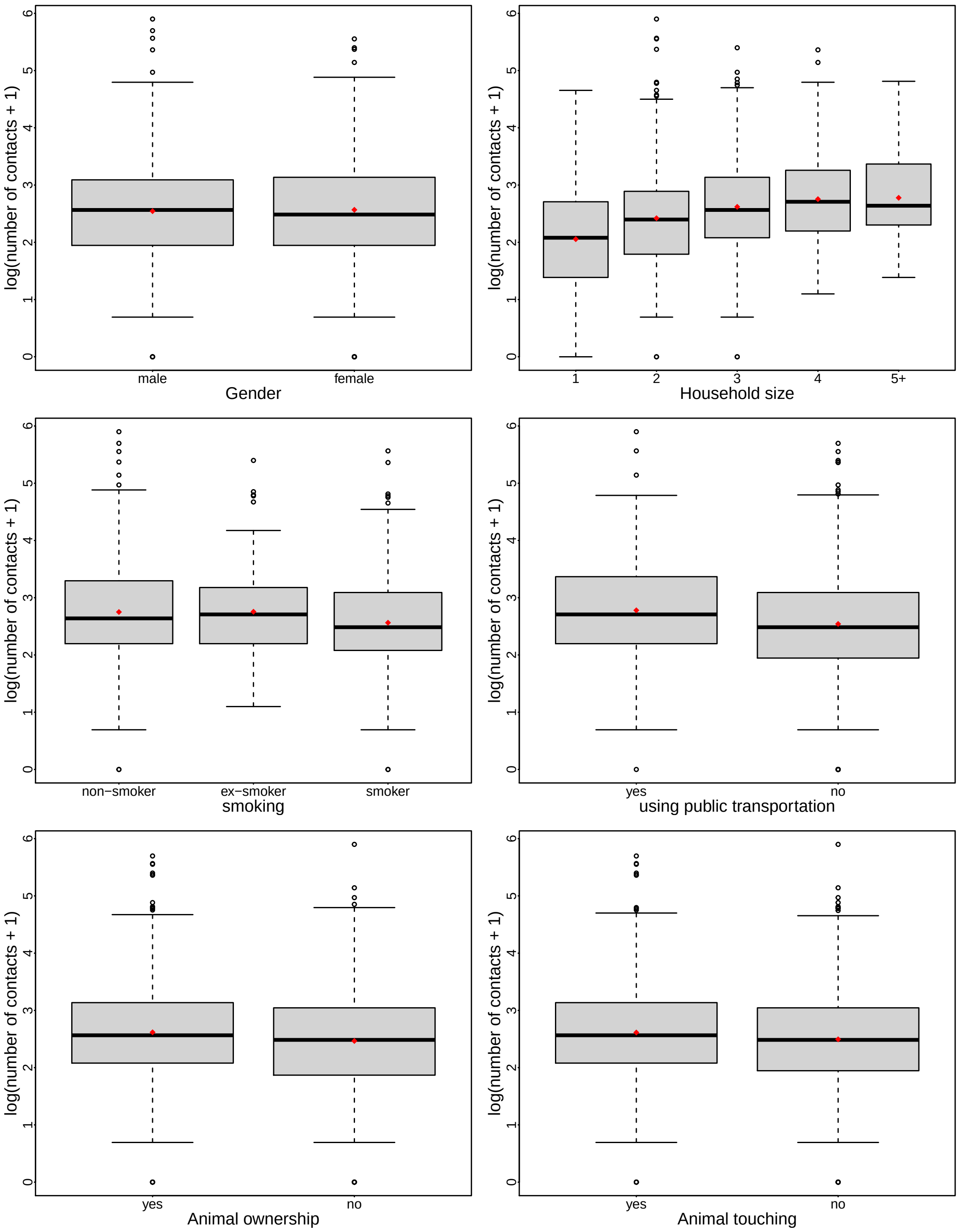

Figure S5: Socio-demographic indicators and the number of contacts. The red dots represent mean values.

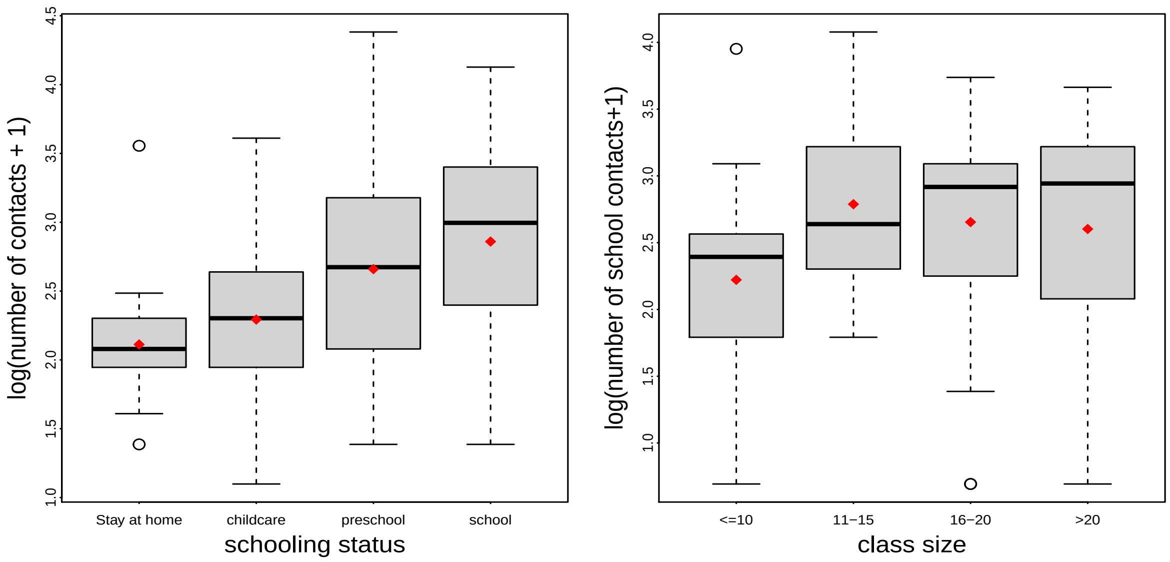

Figure S6: The number of all contacts by different schooling status and the number of school contacts by class sizes

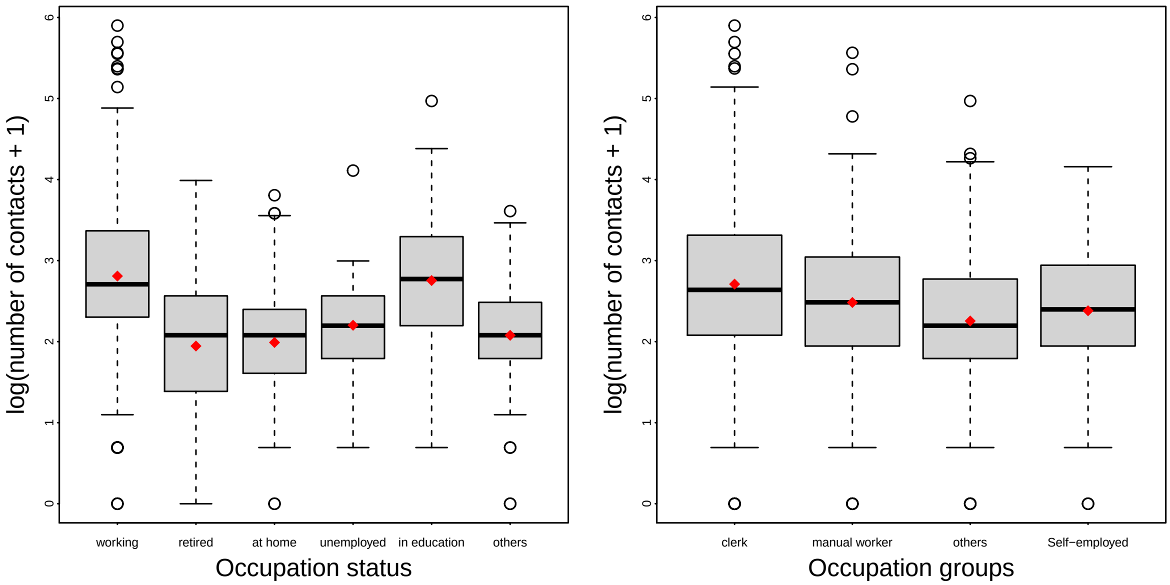

Figure S7: The number of contacts by different occupation statusand occupation groups. Difference in the average number of contacts among occupation statuses and occupation groups are highly significant (P<0.0001, Kruskal-Wallis test).

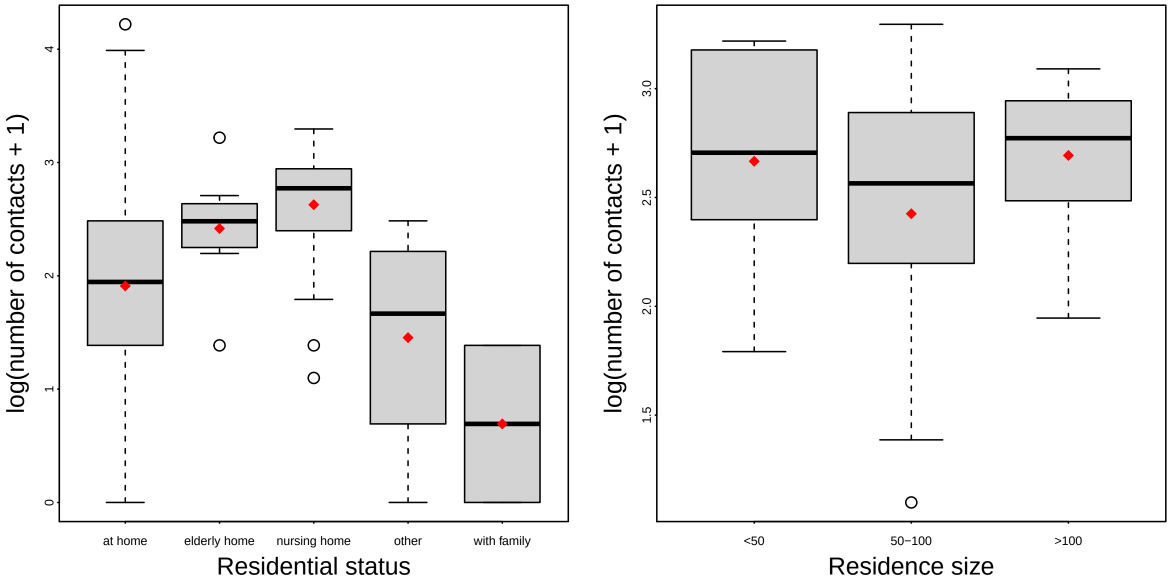

Figure S8: The number of contacts of the elderly by different residential status and residence size. Difference in the average number of contacts is highly significant among residential statuses (P<0.0001, Kruskal-Wallis test), but no significant difference among residence sizes (P=0.47, Kruskal-Wallis test).

6 Contact features

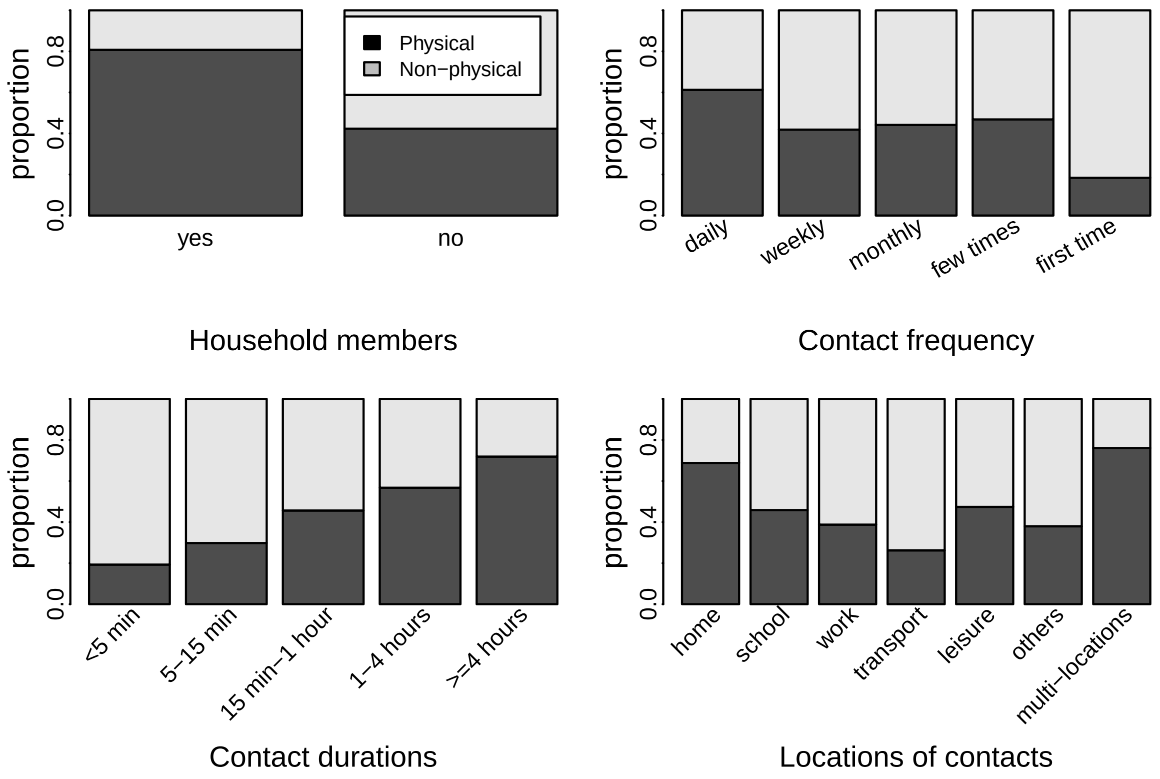

Figure S9: The proportion of physical/non-physical contacts, by household members, frequency, duration, and location

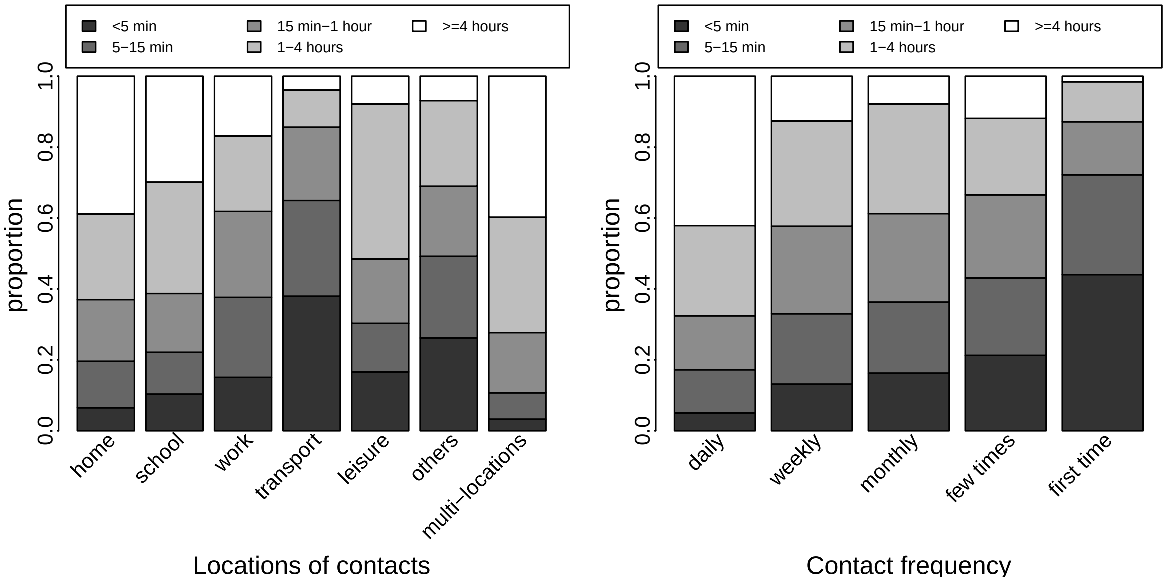

Figure S10: Proportion of contact duration by locations (left) and by contact frequency (right)

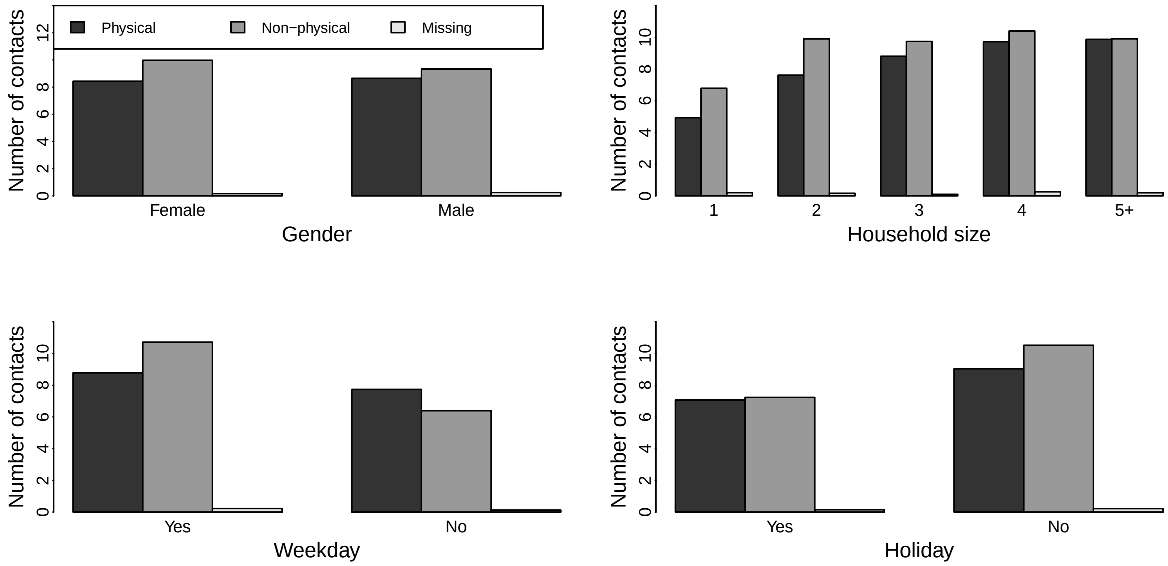

Figure S11: Frequency of physical/non-physical contacts by various factors
