# Supplementary material for: Close contact infection dynamics over time: insights from a second large-scale social contact survey in Flanders, Belgium, in 2010-2011
Source: BMC Infect Dis. 2021 Mar 18;21:274. doi: 10.1186/s12879-021-05949-4 (PMC7971398; doi:10.1186/s12879-021-05949-4)
Supplement: Supplementary file 2 — Additional file 2 Data mining techniques [file 12879_2021_5949_MOESM2_ESM.pdf]

Additional file 2

1 Data mining techniques

1.1 Association rules

Association rule analysis is one of unsupervised learning techniques, with the goal to find the possible relationships between variables or lists of variables in a dataset. We denote a association rule by $X \Rightarrow Y$, where X and Y are lists of variables with X being called "antecedent" and Y the "consequent". Association rules are selected from the set of all possible rules using measures of interestingness including support, confidence and lift values [1, 2]. As for social contact data, the support of a rule is the proportion of contacts in the data expressing all items in that rule, as shown by the following formula:

$$supp(X \Rightarrow Y) = supp(X \cup Y) = \frac{Frequency(X,Y)}{N},$$

where Frequency(XY) represents the number of contacts that are characterized by all variables in X and Y, and N is the total number of contacts in the data. If a rule has a support value of 0.1, it means that 10% of the total number of contacts are used to construct that rule. Value of support helps to identify the rules worth considering for further measures, e.g. confidence and lift. The confidence of a rule is its support divided by the support of X and defined as:

$$conf(X \Rightarrow Y) = \frac{supp(X \cup Y)}{supp(X)},$$

which can be viewed as an estimate of the conditional probability $P(Y|X)$. One drawback of using confidence as a selection tool for association rules is that more frequent variables in Y appear in a dataset, higher are confidence values. Consequently, higher confidence values should not be confused with high correlation [1, 3]. The lift value, defined as the confidence divided by the support of Y, can be used to overcome the problem. A lift value greater 1 means indicates a positive association between variables in X and variables in Y.

$$lift(X \Rightarrow Y) = \frac{supp(X \cup Y)}{supp(X)\ supp(Y)} = \frac{conf(X \Rightarrow Y)}{supp(Y)},$$

1.2 Clustering methods

Clustering data with continuous and categorical variables is performed using Gower distance, calculated in one line using the daisy function in R package "Cluster" [4]. For each variable type, a particular distance metric that works well for that type is used and scaled to fall between 0 and 1. Then, a linear combination using user-specified weights (most simply an average) is calculated to create the final distance matrix. Due to positive skew in contacts at different locations, a log transformation is applied for these variables. The optimal number of clusters is chosen using Sihouette witdth which measure how similar an observation is to its own cluster compared its closet neighboring cluster.

2 Results

2.1 Association rules

Table S1: the strongest association rules according to the lift value, for the following right hand side (rhs) characteristics: (non-)physical contact, long contact and frequent contact; support (X $\Rightarrow$ Y), confidence(X $\Rightarrow$ Y) and lift(X $\Rightarrow$ Y) are provided for each rule

**Y (rhs)**	**X (lhs)**	**Support**	**Confidence**	**Lift**
Physical contact	frequency: daily, duration:> 4 hours	0.10	0.74	1.61
	duration: > 4 hours	0.14	0.71	1.54
	home	0.10	0.71	1.51
Non-physical contact	duration: 0-5 min, hh member: N	0.12	0.81	1.55
	duration: 0-5 min	0.12	0.80	1.54
	holiday: N, duration: 5-15min	0.12	0.70	1.35
	duration: 5-15 min, hh member: N	0.12	0.70	1.35
Long contact (> 4 hours)	frequency: daily, touch: Y, holiday: N, week: weekday	0.14	0.70	3.53
Frequent contact (daily)	hh member: Y	0.11	0.90	2.79
	touch: Y, duration: > 4 hours	0.10	0.71	2.19
	duration: > 4 hours, week: weekday	0.10	0.71	2.19
	holiday: N, duration: > 4 hours	0.11	0.70	2.16

2.2 Clustering methods

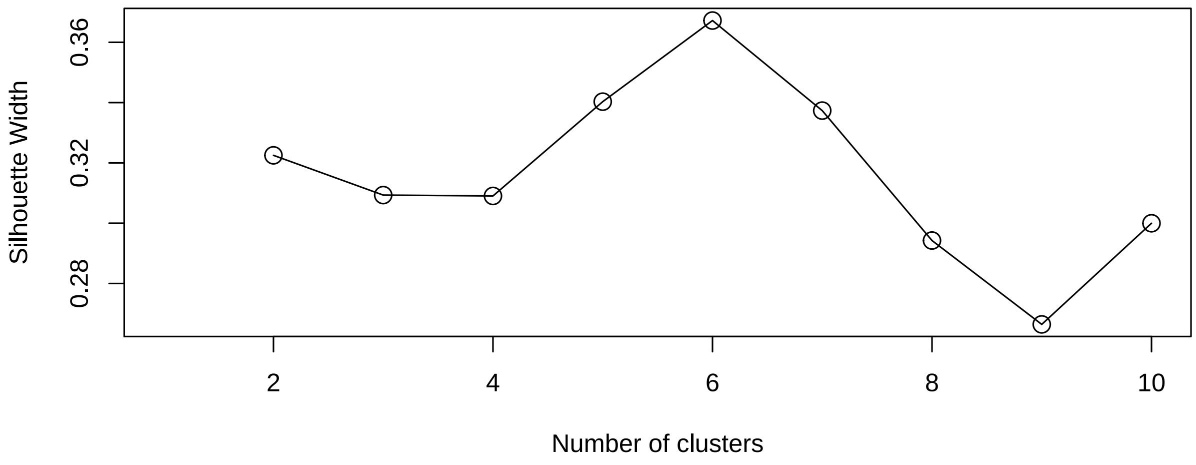

Figure S1: The number of clusters vs the total within-clusters sum of squares

.

Table S2: Detailed characteristics of clusters. In each cell, mean is used for continuous variables and frequency is used for binary variables.

Cluster	home	work	school	leisure	other	transport	Age	types of day		Holiday		Size
								weekday	weekend	No	Yes	
**1**	3.13	0.49	0.08	1.18	3.62	0.35	51.48	443		443		443
**2**	4.01	0.06	14.46	0.92	2.21	0.70	9.30	215		212	3	215
**3**	3.35	3.18	0.07	4.28	4.36	0.37	36.19		257	257		257
**4**	2.49	27.65	0.20	2.30	1.29	0.54	38.91	359		356	3	359
**5**	3.43	1.39	0	2.91	3.69	0.44	36.82		151		151	151
**6**	3.20	6.52	0.12	1.39	3.69	0.52	41.70	280			280	280

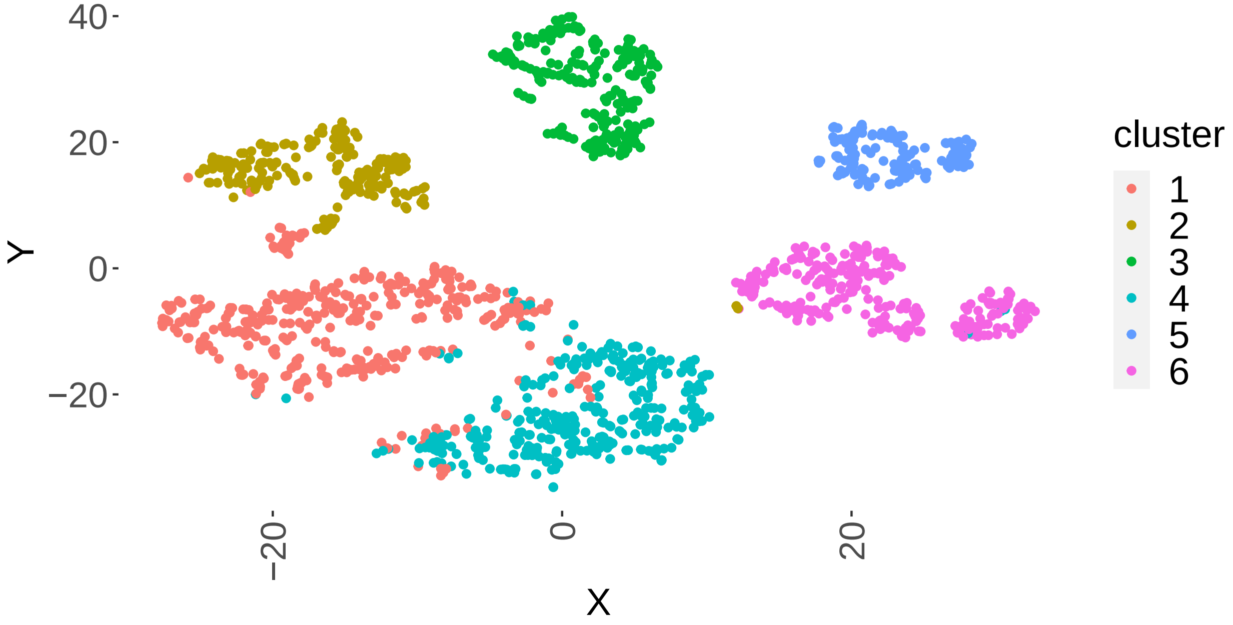

Figure S2: The cluster plots of survey participants using the T-distributed Stochastic Neighbor Embedding technique (t-SNE) for visualization.

References

[1] L. Breiman. Random forests. *Machine learning*, 45(1):5–32, 2001.

[2] L. Breiman. *Classification and regression trees*. Routledge, 2017.

[3] N. Hens, N. Goeyvaerts, M. Aerts, Z. Shkedy, P. Van Damme, and P. Beutels. Mining social mixing patterns for infectious disease models based on a two-day population survey in Belgium. *BMC infectious diseases*, 9(1):1, 2009.

[4] M. Maechler et al. Finding groups in Data: Cluster analysis extended Rousseeuw et. *R Package. version 2.0*, 6, 2018.
