# Supplementary material for: Close contact infection dynamics over time: insights from a second large-scale social contact survey in Flanders, Belgium, in 2010-2011
Source: BMC Infect Dis. 2021 Mar 18;21:274. doi: 10.1186/s12879-021-05949-4 (PMC7971398; doi:10.1186/s12879-021-05949-4)
Supplement: Supplementary file 3 — Additional file 3 Supplementary results of the 2006 and 2010-2011 social contact survey data analyses [file 12879_2021_5949_MOESM3_ESM.pdf]

Additional file 3

1 Results for the analysis of 2010-2011 contact survey data

Model selection

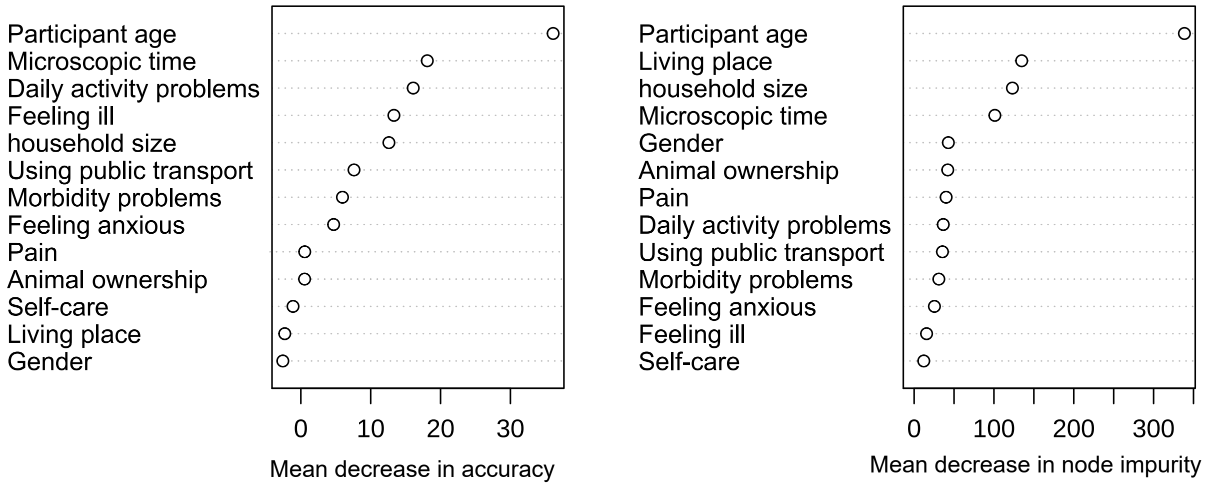

Figure S1: Variable importance calculated by the random forest

Table S1: Model selection based on likelihood ratio test (LRT). DF is the degree of freedom.

	DF	AIC	LRT	P value
Age: Microscopic time	45	12761.01	125.47	<0.001
Gender	1	12723.62	0.07	0.79
Household size	5	12738.29	22.75	<0.001
Living place	5	12734.7	19.16	<0.01
Using public transport	2	12741.56	20.01	<0.001
Animal ownership	2	12723.38	1.83	0.40
Feeling ill	1	12739.95	16.40	<0.001
Mobility problems	2	12728.33	6.79	0.03
Feeling anxious	2	12736.6	15.05	<0.001
Self-care	2	12722.12	0.57	0.75
Daily activity problems	2	12744.62	23.07	< 0.001
Pain	2	12728.05	6.50	0.058

The number of contacts of the older people aged 60+ years who are not living in an elderly/nursing home

Table S2: Negative binomial regression for the number of contacts of the elderly people in Flanders Belgium, in 2010-2011

Covariates	Categories	Estimates (SE)	RNC	95%CI
**Socio-demo indicators**				
Age	(60;65] years old*		1	
	(65;70] years old	-0.01 (0.13)	0.99	[0.77; 1.28]
	(70;75] years old	-0.3 (0.13)	0.74	[0.57; 0.96]
	(75;80] years old	-0.52 (0.15)	0.59	[0.44; 0.8]
	(80;85] years old	-0.23 (0.19)	0.79	[0.54; 1.16]
	(85;90) years old	-1.38 (0.28)	0.25	[0.15; 0.43]
Gender	Female*		1	
	Male	0.25 (0.10)	1.29	[1.05; 1.58]
Having children	No*		1	
	Yes	0.05 (0.25)	1.05	[0.65; 1.71]
Having grandchildren	No*		1	
	Yes	-0.09 (0.19)	0.91	[0.63; 1.32]
**Health indicators**				
Anxiety	no anxious*		1	
	moderate/very anxious	-0.12 (0.15)	0.89	[0.67; 1.18]
	missing	-1.65 (0.79)	0.19	[0.04; 0.89]
Daily activity	no problems*		1	
	some problems/unable	-0.39 (0.15)	0.68	[0.51; 0.91]
	missing	0.52 (1.00)	1.67	[0.23; 12.00]
**Smoking and drinking behavior**				
Smoking	Smoker*		1	
	Ex-smoker	0.46 (0.15)	1.58	[1.18; 2.13]
	Non-smoker	0.41 (0.15)	1.51	[1.13; 2.02]
	unknown	-0.41 (0.49)	0.66	[0.26; 1.72]
Often drinking alcohol	Yes*		1	
	No	-0.20 (0.12)	0.82	[0.65; 1.03]
	Unknown	0.46 (0.48)	1.59	[0.62; 4.06]
**Time indicators**				
Microscopic time	Regular- weekdays*		1	
	Holiday- weekdays	0.22 (0.12)	1.25	[0.99; 1.56]
	Regular- weekend	0.34 (0.14)	1.40	[1.07; 1.85]
	Holiday- weekend	-0.04 (0.19)	0.96	[0.66; 1.40]

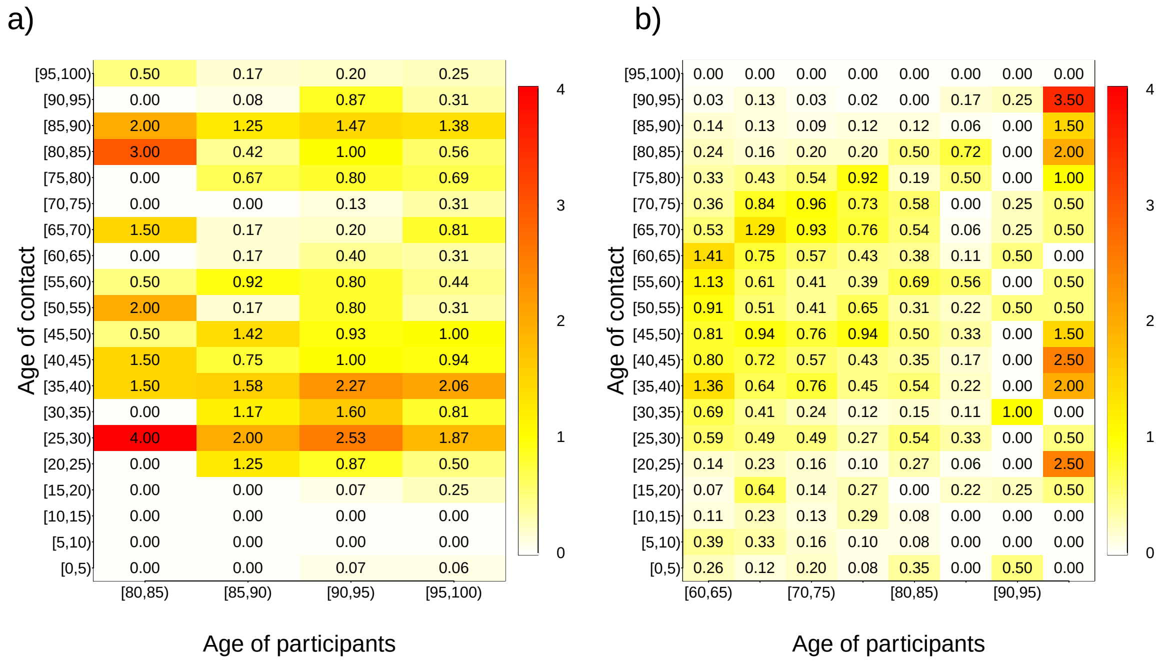

Figure S2: Average number of contacts of people living in an elderly/nursing home (a) and people aged 60+ years living at home (b)

Projected infection risk by age and gender of participants, based on the estimated contact rates

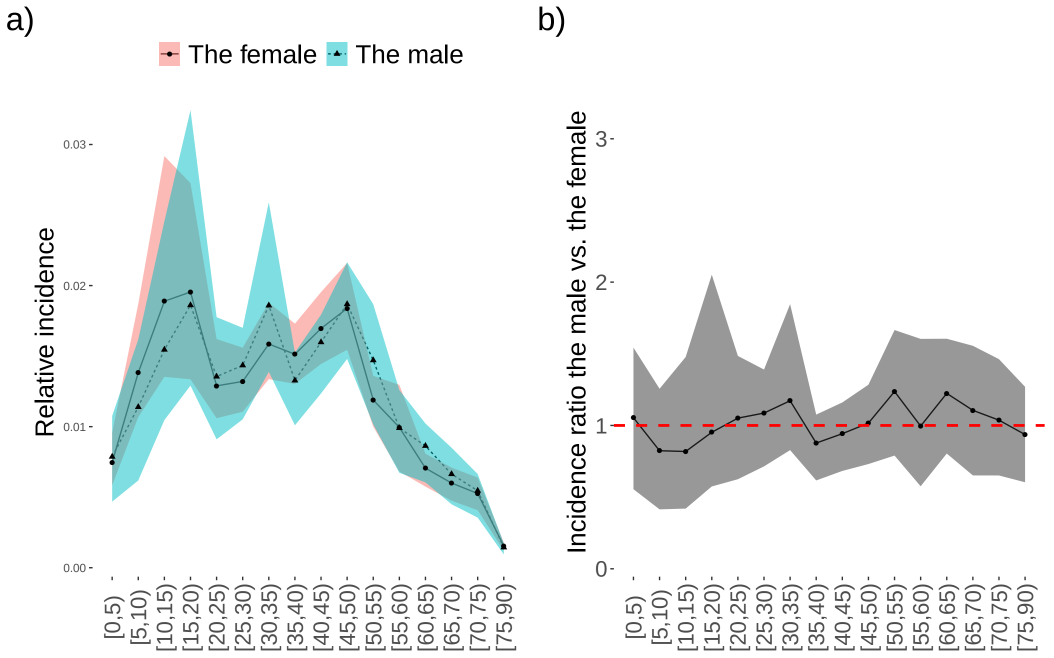

Figure S3: Relative incidence (a) and relative incidence ratio (b)

Modelling the age-specific number of contacts

GAMLSS model

$$\begin{cases} \log(m_{ij}) = \sum_{i=1}^{a} \sum_{j=1}^{a} \beta_{ijs} x_{1it} x_{2jt} \\ \log(\kappa_{ij}) = \gamma_0 + \sum_{j=1}^{a} \gamma_j x_{2jt} \end{cases} \quad (1)$$

$$x_{1it} = \begin{cases} 1 & \text{for participants of age class } i \\ 0 & \text{otherwise} \end{cases}$$

$$x_{2jt} = \begin{cases} 1 & \text{for contacts of age class } j \\ 0 & \text{otherwise} \end{cases}$$

where β_{ijs} and γ_j are regression coefficients associated to interaction terms $x_{1it}x_{2jt}$ (for all $i$and j) of the mean and over-dispersion in the model 1, respectively. The elements $m_{ij} = \exp(\beta_{ij})$ are the number of contacts per day made by participants of age class i with people of age class j.

Comparison of the basic reproduction number

The element of $\boldsymbol{G}$ (i.e., g_{ij}) indicates the expected number of secondary infections in age class i through the introduction of a primary infectious individual of age class j into a fully susceptible population. The next generation matrix is defined as:

$$\boldsymbol{G} = \frac{ND}{L} qC, \quad (2)$$

where N is the population size, D is the mean duration of infectiousness, L is the life expectancy, C is the social contact matrix and q is the proportionality factor. We estimate the relative change in basic reproduction number, as RR_0, between two groups:

$$RR_0 = \frac{Max\ eigen\ value(G_1)}{Max\ eigen\ value(G_{2'})} = \frac{Max\ eigen\ value(C_1)}{Max\ eigen\ value(C_{2'})}, \quad (3)$$

where indices 1 and 2 refer to the contacts recorded during the weekend and the weekday, or during the holiday and the regular period, respectively. The result will be presented with 95% bootstrap-based percentile CIs.

2 Results for the analysis of 2006 and 2010-2011 contact survey data

Age distribution and number of contacts by age groups in 2 social contacts surveys

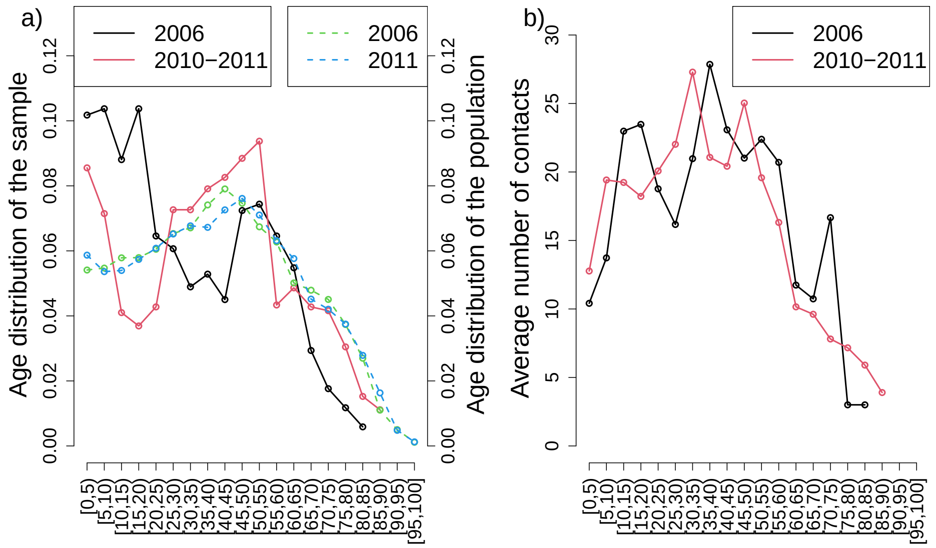

Figure S4: The proportion of surveyed participants (solid lines) and Flemish population by 5 year age groups (dotted lines) (a) and the Flemish population-adjusted average number of contacts in the surveys in 2006 and 2010-2011 (b).

Investigating potential risk factor for the number of contacts

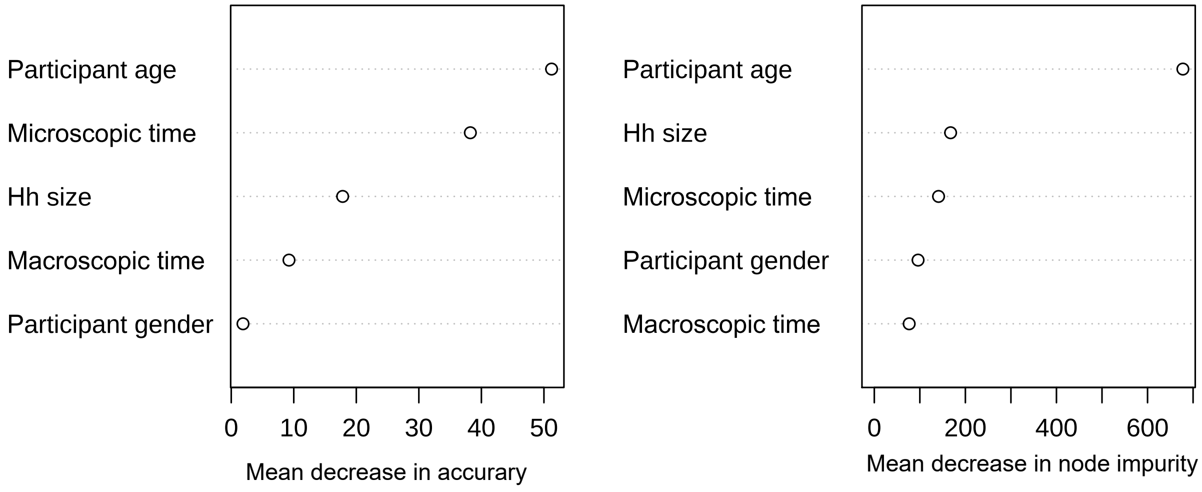

Figure S5: Variable importance calculated by the random forest

Table S3: Variable selection for the mean and over-dispersion parameters of the GAMLSS of the total number of contacts tested by likelihood ratio test.

	Mean structure				Dispersion structure			
	**DF**	**AIC**	**LRT**	**P-value**	**DF**	**AIC**	**LRT**	**P-value**
Household size	5	16477.85	31.29	8.21E-06	5	16477.94	31.38	7.86E-06
Age group: Microscopic time	26	16539.62	135.06	1.07E-16	26	16478.31	73.74	1.86E-06
Age group: Macroscopic time	13	16479.16	48.59	5.18E-06	13	16469.28	38.72	0.000221
Microscopic time: Macroscopic time	2	16481.63	29.07	4.87E-07	2	16464.04	11.48	0.003214

Table S4: GAMLSS model for the total number of contacts in the aggregated data from the 2006 survey and the 2010 survey. Coefficient estimates are displayed with Standard Error (SE), Relative Number of Contacts (RNC) and 95% CI of RNC.

**Categories**	**Covariates**	**Estimate (SE)**	**RNC**	**95% CI**
Age				
	[0; 5) years*		1	
	[5; 10) years	-0.1(0.22)	0.90	[0.59;1.4]
	[10; 15) years	0.69(0.26)	1.99	**[1.2;3.3]**
	[15; 20) years	0.37(0.24)	1.44	[0.91;2.3]
	[20; 25) years	-0.23(0.22)	0.79	[0.52;1.21]
	[25; 30) years	-0.31(0.22)	0.73	[0.47;1.14]
	[30; 35) years	0.37(0.23)	1.44	[0.92;2.28]
	[35; 40) years	0.06(0.24)	1.07	[0.67;1.71]
	[40; 45) years	-0.19(0.21)	0.83	[0.54;1.26]
	[45; 50) years	0.85(0.28)	2.34	**[1.34;4.07]**
	[50; 55) years	0.32(0.24)	1.38	[0.85;2.23]
	[55; 60) years	0.01(0.27)	1.01	[0.60;1.70]
	[60; 65) years	-0.66(0.25)	0.52	**[0.32;0.85]**
Household size				
	1*		1	
	2	0.29(0.07)	1.33	**[1.16;1.52]**
	3	0.23(0.07)	1.26	**[1.09;1.46]**
	4	0.23(0.07)	1.26	**[1.09;1.45]**
	5	0.39(0.08)	1.47	**[1.26;1.72]**
	missing	-0.16(0.37)	0.86	[0.42;1.76]
Microscopic time				
	Regular- weekdays*		1	
	Holiday- weekdays	0.15(0.29)	1.16	[0.66;2.05]
	Weekend	-1.10(0.16)	0.33	**[0.24;0.45]**
Macroscopic time				
	Year 2006		1	
	Year 2010/2011	-0.45(0.17)	0.64	**[0.46;0.89]**
Age: Microscopic time				
	[5; 10) years: Holiday- weekdays	-0.56(0.30)	0.57	[0.32;1.02]
	[10; 15) years: Holiday- weekdays	-0.68(0.29)	0.51	**[0.29;0.88]**
	[15; 20) years: Holiday- weekdays	-0.92(0.30)	0.4	**[0.22;0.71]**
	[20; 25) years: Holiday- weekdays	-0.16(0.30)	0.85	[0.47;1.53]
	[25; 30) years: Holiday- weekdays	0.19(0.33)	1.21	[0.64;2.31]
	[30; 35) years: Holiday- weekdays	-0.99(0.29)	0.37	**[0.21;0.65]**
	[35; 40) years: Holiday- weekdays	-0.23(0.3)	0.8	[0.44;1.43]
	[40; 45) years: Holiday- weekdays	0.24(0.34)	1.27	[0.65;2.49]
	[45; 50) years: Holiday- weekdays	-0.05(0.33)	0.95	[0.50;1.81]
	[50; 55) years: Holiday- weekdays	-0.01(0.30)	0.99	[0.55;1.77]
	[55; 60) years: Holiday- weekdays	-0.1(0.32)	0.9	[0.48;1.70]
	[60; 65) years: Holiday- weekdays	0.16(0.30)	1.18	[0.65;2.13]
	[5; 10) years: Weekend	0.29(0.19)	1.33	[0.92;1.94]
	[10; 15) years: Weekend	0.002(0.2)	1.00	[0.67;1.49]
	[15; 20) years: Weekend	0.12(0.19)	1.12	[0.77;1.64]

Table S4

Categories	Covariates	Estimate (SE)	RNC	95% CI
		Continued previous page		
	[20; 25) years: Weekend	0.72(0.21)	2.06	**[1.37;3.10]**
	[25; 30) years: Weekend	0.16(0.2)	1.17	[0.8;1.72]
	[30; 35) years: Weekend	-0.14(0.2)	0.87	[0.58;1.29]
	[35; 40) years: Weekend	0.08(0.21)	1.08	[0.72;1.63]
	[40; 45) years: Weekend	-0.03(0.18)	0.97	[0.68;1.4]
	[45; 50) years: Weekend	0.37(0.23)	1.45	[0.92;2.29]
	[50; 55) years: Weekend	0.18(0.19)	1.20	[0.82;1.74]
	[55; 60) years: Weekend	0.43(0.21)	1.54	**[1.02;2.34]**
	[60; 65) years: Weekend	0.96(0.22)	2.60	[1.70;4.00]
Age: Macroscopic time				
	[5; 10) years: Year 2010/2011	0.45(0.21)	1.57	**[1.04;2.35]**
	[10; 15) years: Year 2010/2011	-0.22(0.24)	0.8	[0.5;1.28]
	[15; 20) years: Year 2010/2011	0.003(0.22)	1.00	[0.65;1.54]
	[20; 25) years: Year 2010/2011	0.47(0.21)	1.60	**[1.06;2.41]**
	[25; 30) years: Year 2010/2011	0.79(0.21)	2.2	**[1.47;3.31]**
	[30; 35) years: Year 2010/2011	0.49(0.21)	1.63	**[1.07;2.48]**
	[35; 40) years: Year 2010/2011	0.44(0.23)	1.56	[0.98;2.46]
	[40; 45) years: Year 2010/2011	0.65(0.20)	1.92	**[1.29;2.85]**
	[45; 50) years: Year 2010/2011	-0.27(0.29)	0.76	[0.43;1.34]
	[50; 55) years: Year 2010/2011	-0.01(0.22)	0.99	[0.64;1.52]
	[55; 60) years: Year 2010/2011	0.13(0.25)	1.14	[0.70;1.85]
	[60; 65) years: Year 2010/2011	0.16(0.24)	1.18	[0.73;1.89]
Macroscopic time: Microscopic time				
	Year 2010/2011: Holiday- weekdays	-0.34(0.16)	0.71	**[0.52;0.97]**
	Year 2010/2011: Weekend	0.42(0.10)	1.52	**[1.25;1.85]**

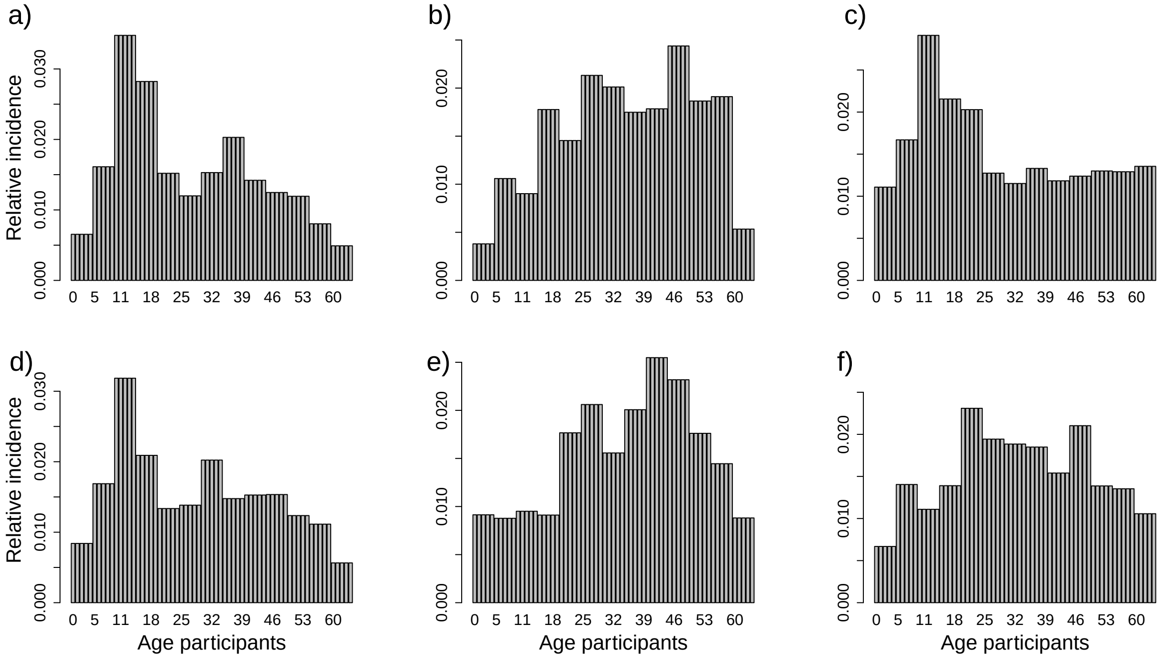

Figure S6: Relative incidence during regular weekday (a,d), holiday weekday (b,e) and weekend (c,f). The 1st row stands for the 2006 survey data and the 2nd row for the 2010-2011 survey data.

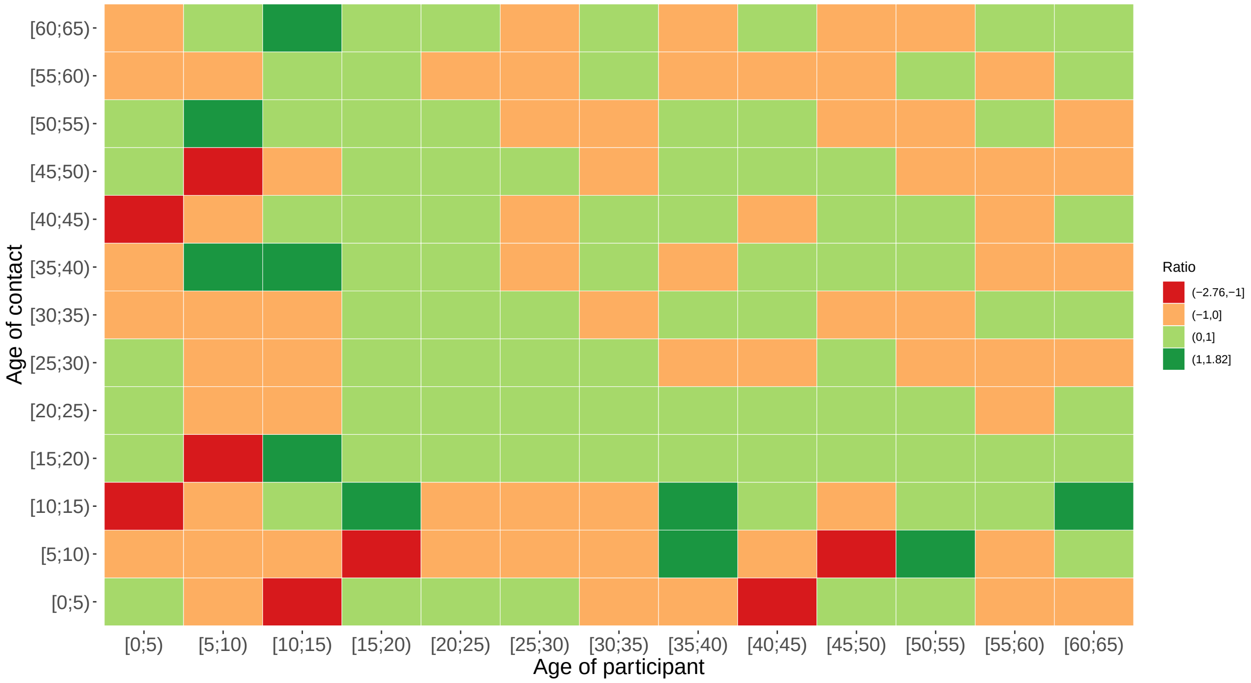

Figure S7: Comparison of contact matrices that are estimated from the 2006 and 2011 social contact surveys in Flanders during weekday-regular. The cells in this this matrix shows logarithm of transmission rate ratio, where significant results are shown by stars. CI are obtained after the Bonferroni correction at an initial significance level of 0.05.

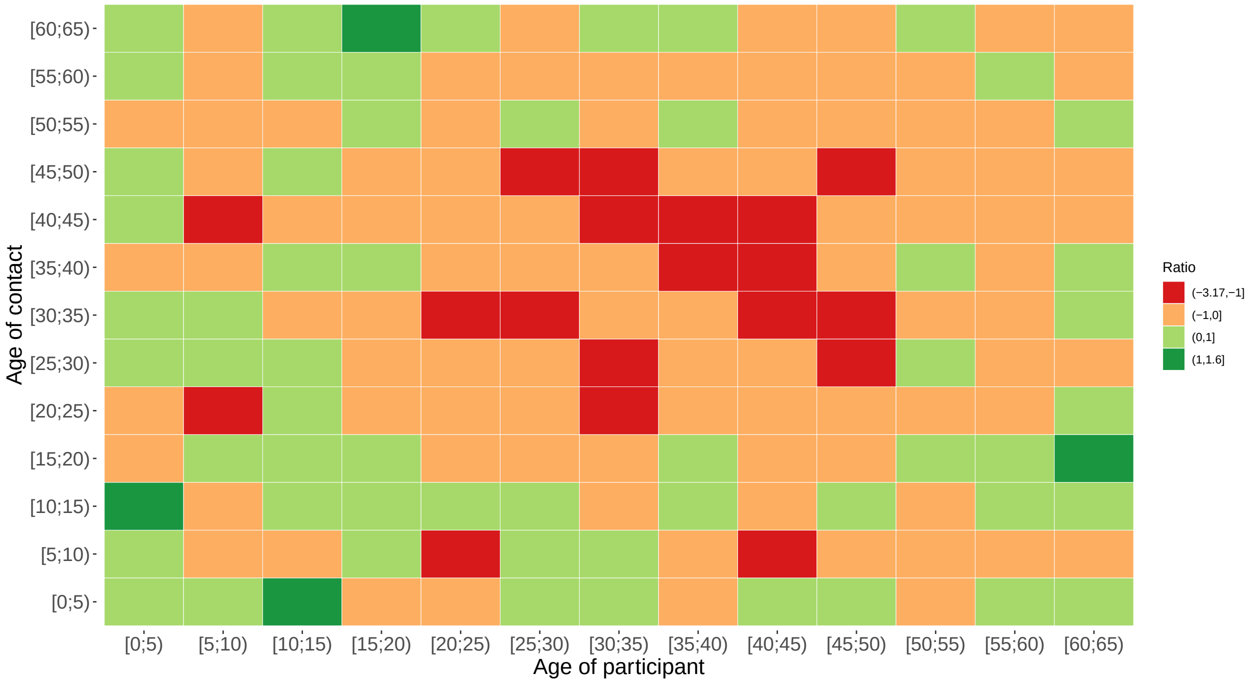

Figure S8: Comparison of contact matrices that are estimated from the 2006 and 2011 social contact surveys in Flanders during weekend. The cells in this matrix shows logarithm of transmission rate ratio, where significant results are shown by stars. CI are obtained after the Bonferroni correction at an initial significance level of 0.05.

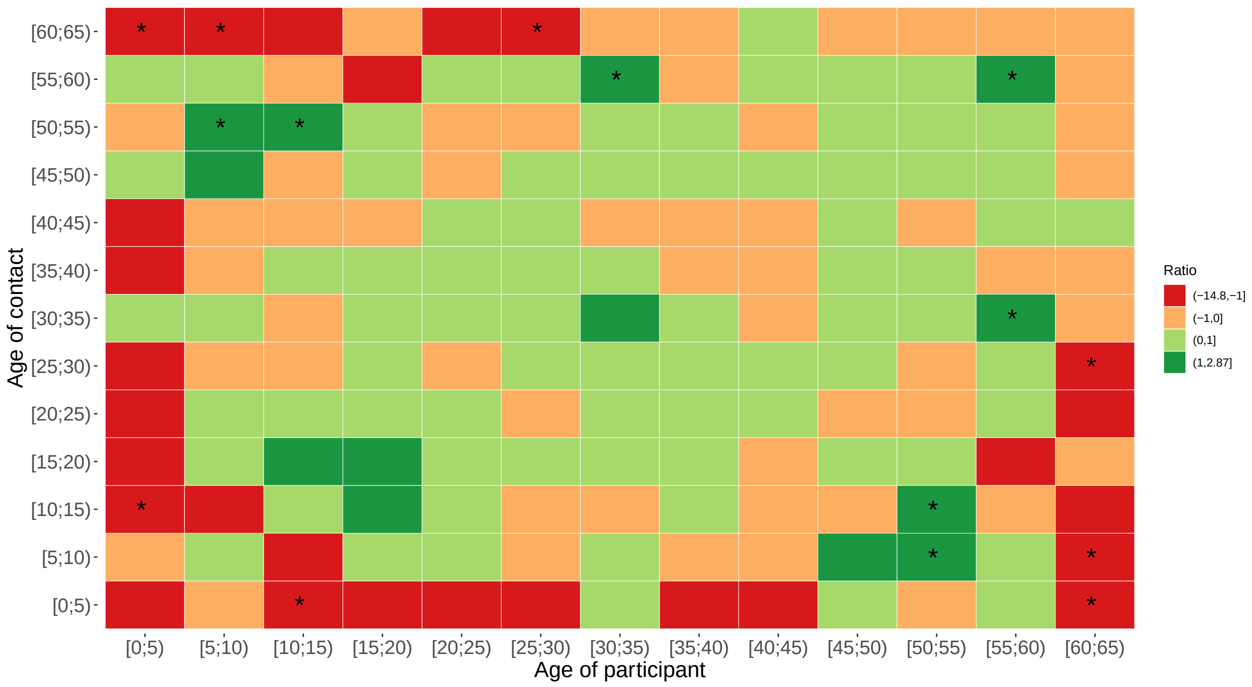

Figure S9: Comparison of contact matrices that are estimated from the 2006 and 2011 social contact surveys in Flanders during holiday- weekdays. The cells in this this matrix shows logarithm of transmission rate ratio, where significant results are shown by stars. CI are obtained after the Bonferroni correction at an initial significance level of 0.05.

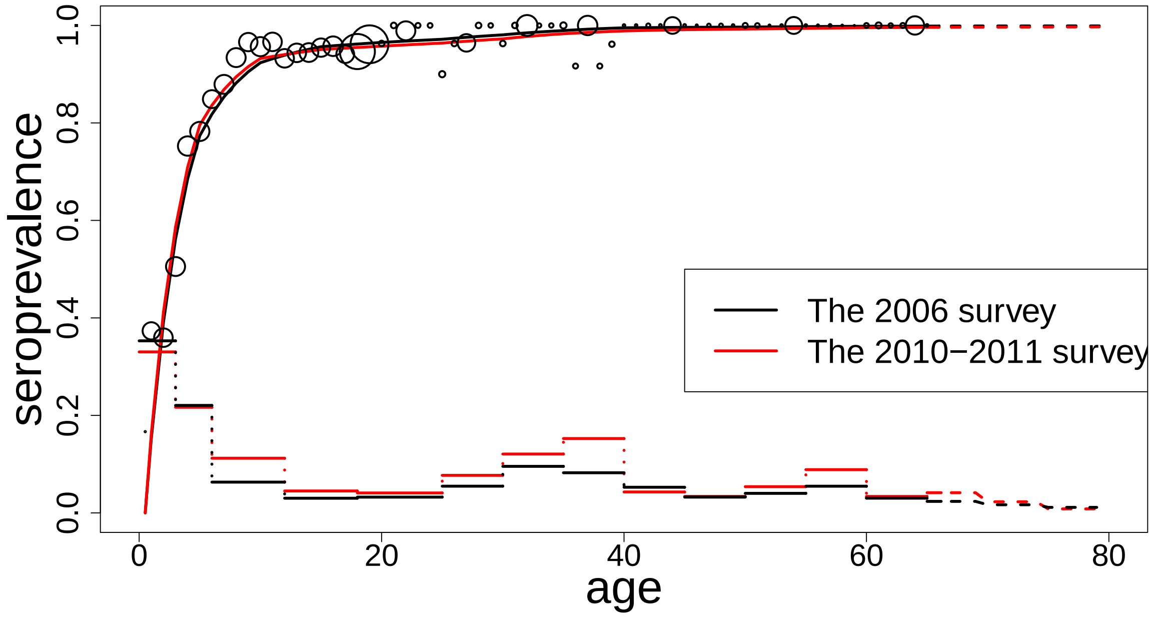

Figure S10: Sero-prevalence obtained by contact matrices in 2006 and 2010-2011
